# Repeated bronchoscopy in health and obstructive lung disease: is the airway microbiome stable?

**DOI:** 10.1186/s12890-021-01687-0

**Published:** 2021-11-02

**Authors:** Rune Nielsen, Yaxin Xue, Inge Jonassen, Ingvild Haaland, Øyvind Kommedal, Harald G. Wiker, Christine Drengenes, Per S. Bakke, Tomas M. L. Eagan

**Affiliations:** 1 Department of Clinical Science, Faculty of Medicine, University of Bergen, Postboks 7804, 5020 Bergen, Norway; 2 Department of Thoracic Medicine, Haukeland University Hospital, Bergen, Norway; 3 Computational Biology Unit, Department of Informatics, University of Bergen, Bergen, Norway; 4 Department of Microbiology, Haukeland University Hospital, Bergen, Norway

**Keywords:** Microbiota, Microbiome, COPD, Repeatability, Reliability, Bronchoscopy, 16S rRNA, Next generation sequencing

## Abstract

**Objective:**

Little is known concerning the stability of the lower airway microbiome. We have compared the microbiota identified by repeated bronchoscopy in healthy subjects and patients with ostructive lung diseaseases (OLD).

**Methods:**

21 healthy controls and 41 patients with OLD completed two bronchoscopies. In addition to negative controls (NCS) and oral wash (OW) samples, we gathered protected bronchoalveolar lavage in two fractions (PBAL1 and PBAL2) and protected specimen brushes (PSB). After DNA extraction, we amplified the V3V4 region of the 16S rRNA gene, and performed paired-end sequencing (Illumina MiSeq). Initial bioinformatic processing was carried out in the QIIME-2 pipeline, identifying amplicon sequence variants (ASVs) with the DADA2 algorithm. Potentially contaminating ASVs were identified and removed using the decontam package in R and the sequenced NCS.

**Results:**

A final table of 551 ASVs consisted of 19 × 10^6^ sequences. Alpha diversity was lower in the second exam for OW samples, and borderline lower for PBAL1, with larger differences in subjects not having received intercurrent antibiotics. Permutational tests of beta diversity indicated that within-individual changes were significantly lower than between-individual changes. A non-parametric trend test showed that differences in composition between the two exams (beta diversity) were largest in the PSBs, and that these differences followed a pattern of PSB > PBAL2 > PBAL1 > OW. Time between procedures was not associated with increased diversity.

**Conclusion:**

The airways microbiota varied between examinations. However, there is compositional microbiota stability within a person, beyond that of chance, supporting the notion of a transient airways microbiota with a possibly more stable individual core microbiome.

**Supplementary Information:**

The online version contains supplementary material available at 10.1186/s12890-021-01687-0.

## Introduction

Microbiota studies of the lungs are subject to specific challenges. The biomass in the lower airways is low [1, 2] and consequently prone to the influence of contaminants both during the sampling procedure and laboratory processing [1, 3]. To counter this, direct sampling from the lower airways are necessary. The current gold standard for sampling is by bronchoscopy, which invariably is somewhat invasive and comes with some discomfort. Thus large scale studies with bronchoscopy performed solely for research are still scant. Furthermore, results in some fields seem to be partly diverging—for instance for chronic obstructive pulmonary disease (COPD) there are studies pointing to both a more and less diverse microbiota with the disease. One hypothesis might be that these differences result from our personal airway microbiome being in constant flux.

Little is truly known about the stability of the airway microbiota over time, but at least three studies have published results on repeat bronchoscopies [4–6]. They were all intervention studies, but one of them included a control group that also was subject to repeated bronchoscopy [4]. Segal et al. reported the effect on microbiota in a randomized controlled trial on the effect of azithromycin on smokers with emphysema [4]. In a control group of 10 individuals they reported no significant changes, but the study did not aim to specifically investigate the degree of stability of the microbiome in individuals, and did not visualize the changes in the microbial composition of the airway samples of the individual participants in the control group.

A few studies based on sputum samples have also looked at the repeatability [7, 8], but the validity of these as a proxy for the airway microbiota is questionable, since there will always be contamination from the high-biomass oral cavity. As far as we know no sputum study have looked at healthy subjects. Furthermore, sputum studies cannot accurately locate the sampling site within the airways.

The aim of the current study was to investigate how the microbiota changes over time in subjects with and without obstructive lung disease based on repeated lower airways sampling, and assess the effect of time, lung function (FEV_1_ in percent predicted), use of inhaled corticosteroids (ICS) and intercurrent antibiotics.

## Methods

The initial protocol of the Bergen COPD microbiome study (short name "MicroCOPD") has been previously published [9]. All participants provided written, informed consent. The study was conducted in accordance with the Helsinki declaration, and was approved by the Regional Ethics Committee of Western Norway (Project Number 2011/1307).

### Participants and data collection

249 subjects participated with at least one bronchoscopy in MicroCOPD study [9, 10]. Participants were eligible to be invited to a second bronchoscopy provided they had been able to cooperate and no serious adverse event had taken place in the first procedure. 62 study participants completed a second bronchoscopy (21 healthy controls, 40 COPD patients and 1 asthma patient). Two COPD patients lacked adequate negative control samples from the second procedure, thus the current study sample included 60 participants. Of these 60 participants, 7 healthy controls and 4 COPD patients underwent a third bronchoscopy.

COPD and asthma were diagnosed based on a combination of medical history and evaluation of post-bronchodilator spirometry, computed tomography (CT) scans of the lungs and clinical examination. There was no lung or airways disease in the medical history, or signs of such from the clinical examination, lung function tests or CT scans of the control subjects.

Subjects that had oxygen saturation below 90% despite oxygen supplementation, hypercapnia, increased bleeding risks, or cardiac risk factors for bronchoscopy were excluded. If participants had received antibiotics or oral corticosteroids the preceding 14 days, or showed signs of an ongoing respiratory symptom exacerbation, participation was postponed.

Before the bronchoscopy, we collected oral wash (OW) samples by letting participants gargle 10 mL sterile phosphate buffered saline (PBS). Participants were offered light sedation (alfentanil), and were also given topical anesthesia (lidocaine) delivered preoperatively and through a spray catheter during intubation. The bronchoscopy was performed with the patient in supine position. Oral access was achieved through a mouth guard. To help avoid contamination no suction was allowed before the carina. During the procedure, we sampled 3 protected sterile brushes from the right lower lobe (rPSB) and 3 from the left upper lobe (lPSB). Protected bronchoalveolar lavage [2] was collected from the right middle lobe by instilling 2 × 50 mL of PSB (PBAL1 and PBAL2) within a sterile inner catheter placed in the working channel, and extracted with the same sterile syringe through which the fluid was instilled. Small-volume lavage was obtained with suction through the working channel of the bronchoscope after instilling 20 mL of PBS into the left upper lobe (SVL). Each clinical visit was accompanied by a separate negative control sample (NCS) with PBS from the same batch that was used in the procedural samples. Thus, in principle there were 2 NCS samples from those individuals that were examined twice, and 3 NCS samples from those individuals that were examined three times. Some of the samples had to be re-run (e.g. low signal or sequencing number), and in these cases also the NCS samples were re-run, thus the total number of NCS samples were 143.

### DNA extraction, PCR and sequencing

The full protocol for the DNA extraction, PCR, and sequencing is publicly available on the protocols.io repository [11]. Briefly, we used a combination of enzymatic and mechanic bacterial cell lysis, followed by DNA purification and 45 cycles of targeted PCR for the V3V4 region of the bacterial 16S rRNA gene. DNA sequencing was performed using paired-end sequencing (2 × 300 cycles) on an Illumina MiSeq sequencer according to the Illumina 16S Metagenomic Sequencing Library Preparation guide (Part no. 15044223 Rev. B).

### Bioinformatics and statistics

Characteristics of participants were evaluated using t-tests, chi squared tests, and non-parametric tests after judging the distribution of the data.

We used the second version of the quantitative insights into microbial ecology (QIIME2) as our main bioinformatics pipeline [12]. Removal of primers, quality control, joining of paired ends, chimera removal, and identification of amplicon sequence variants (ASVs) were performed in one initial command in separate batches for each MiSeq run—*qiime dada2 denoise-paired.* Sequences were truncated at positions where the median phred scores fell below 25 for the forward reads, and below 20 for the reverse reads (identified using the *qiime demux summarize*—command). The large number of ASVs led us to perform an additional chimera removal using the VSEARCH algorithm [13]. Next all samples with less than 1000 sequences, and all ASVs simultaneously present in less than 10 different samples and having less than 2000 sequences (roughly corresponded to 0.005% of sequences) was removed [14], both to exclude samples of low quality and to remove spurious ASVs like undetected chimeras.

For taxonomic classification, we chose the human oral microbiome database (http://www.homd.org/ Version 15.1, downloaded March 8th 2018), using the Naïve Bayes classifier in QIIME2 [15]. ASVs unclassified below kingdom level were removed. We applied the *Decontam* package in *R* to further reduce the potential impact of contamination for low biomass samples [16]. *Decontam* was run with MiSeq runs identified as batches using the “either” option, utilizing both the prevalence-based and frequency-based algorithms with thresholds set at 0.3 for the latter and 0.5 for the former algorithm. Since total DNA concentrations had been measured before loading to the MiSeq by both the PicoGreen and Qubit methods, we performed frequency-based *decontam* analyses stratified by DNA measure method. Control samples were thereafter removed from the resulting merged file. Finally, we manually inspected the 50 most frequent taxa and removed one microbial species that was highly likely to be a contaminant (*Mesorhizobium loti*) [3].

Alpha-diversity (within sample diversity) was estimated in cumulative sum scale (CSS) normalized files where samples with less than 200 sequences were excluded [17], and tested statistical difference in Stata 14 using (paired by individual) non-parametric Wilcoxon signed-rank tests. Beta-diversity was explored through Bray Curtis distances and Weighted UniFrac computed using the PhyloSeq package in *R* and QIIME2, respectively. We performed permutation tests (10,000 random samplings) to assess how often randomly drawn samples were as similar as samples from the same patient. We did not exclude the possibility of drawing the actual pairs in this re-sampling procedure. Associations of beta-diversity between procedure 1 and procedure 2 were compared by multivariable beta regression in Stata 15.

## Results

The characteristics of the study sample are presented in Table 1.

**Table 1:** Characteristics of participants examined two times by bronchoscopy in the MicroCOPD study

	COPD	Controls	Asthma	Comparison of COPD versus control
N	38	21	1	
Men, N (%)	24 (68%)	14 (67%)	1	*p* > 0.05*
Age at exam 1, years, median (IQR)	69.3 (9.1)	67.8 (7.0)	55.2	*p* > 0.05***
Never smoker, N (%)	0	1 (5%)	0	*p* > 0.05*
Current smoker, N (%)	9 (24%)	7 (33%)	0	
Ex-smoker, N (%)	29 (76%)	13 (62%)	1	
Pack years, median (IQR)	30 (22)	24 (16..8)	38	*p* > 0.05***
FEV_1_, % predicted (SD)	55.6 (28.2)	100.1 (14.6)	102.3	*p* < 0.001***
ICS use, N (%)	26 (68%)	1 (5%)	0	*p* < 0.001*
Antibiotic use between exams 1 and 2, N (%)	10 (26.3)	4 (19.1)	0	*p* > 0.05*
Median number of days between exam 1 and 2 (IQR)	139.5 (75)	156 (33)	298	*p* > 0.05***

*IQR* interquartile range, *FEV*_*1*_ forced expiratory volume in 1 s, *ICS* inhaled corticosteroids, *COPD* chronic obstructive pulmonary disease

*Chi-square test

**t-test

***Wilcoxon rank-sum test

**Fig. 1:**
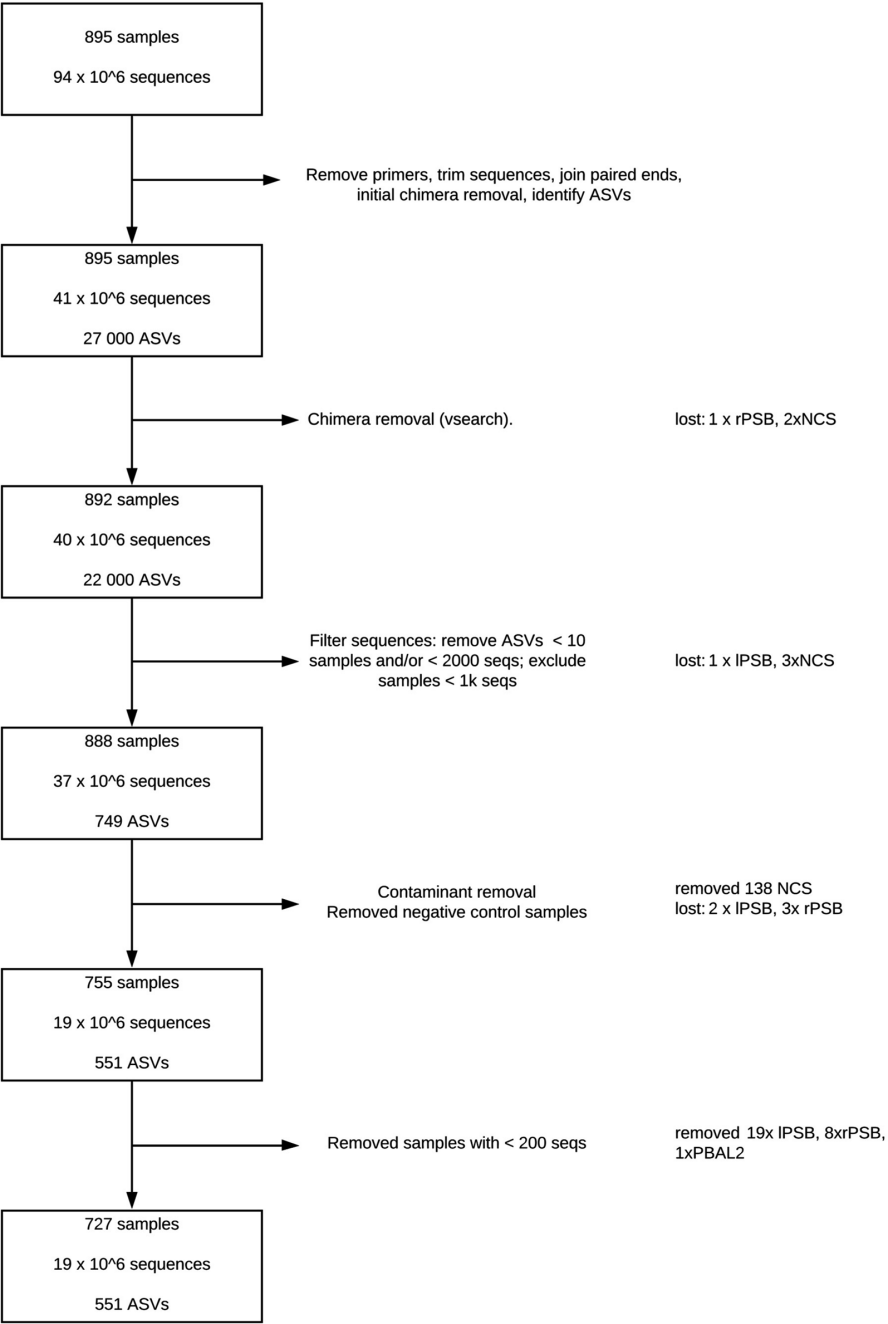
Upstream analyses (bioinformatic processing and clean-up) of samples for the MicroCOPD re-bronchoscopy study. ASV: amplicon sequence variant. PBAL1: first fraction of protected broncho-alveolar lavage. PBAL2: second fraction of protected broncho-alveolar lavage. rPSB: protected specimen brush from right lower lobe. lPSB: protected specimen brush from left upper lobe. NCS: negative control samples

We started out with 895 samples, that were reduced to altoghether 727 samples with 19 million sequences distributed over 551 amplicon sequence variants (ASVs). These upstream analyses are detailed in Fig. 1. The number of samples in exam 1 and 2 varied, but we had paired samples from both exam 1 and 2 for 60 OW, 52 PBAL1, 49 PBAL2, 52 rPSB and 43 lPSB samplings.

**Fig. 2:**
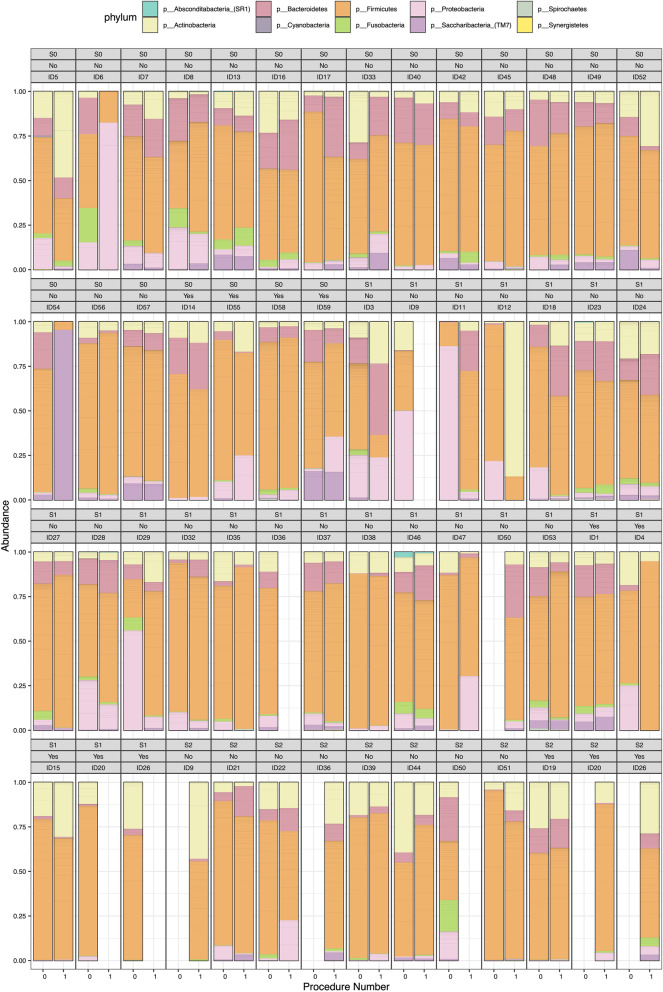
Taxonomic distribution (phylum level) of all amplicon sequence variants in the first fraction of the protected broncho-alveolar lavage samples (PBAL1), shown by participant and procedure number (first column: first bronchoscopy, second column: second bronchoscopy). Ordered by disease stage (first grey box, S0: control subjects, S1: COPD, FEV1 > 50% of predicted, S2: COPD, FEV1 < 50% of predicted and whether subjects have received antibiotics between procedures (second grey box; No or Yes). The third grey box is an anonymous participant identification number

We first compared the taxonomical composition of the PBAL1 at the phylum level for individual participants at exam 1 and exam 2 (Fig. 2). The coupled bars are ordered by use of antibiotics and by FEV_1_ category. No apparent pattern related to use of antibiotics and FEV_1_ category stage was seen by visual examination, however the first and second procedure bear resemblance in most cases. Additional file 1: Figs. S1 and S2 shows the same analyses for other sampling methods and for the genus level for the top 20 ASVs.

After assessing the taxonomical composition we had a look at how the overall microbial composition of samples changed between exam 1 and 2—i.e. alpha and beta diversity. Alpha diversity indicates how rich a specific sample is (the simplest measure is thus the number of different ASVs within a sample). Beta diversity compares the overall composition between two samples.

**Fig. 3:**
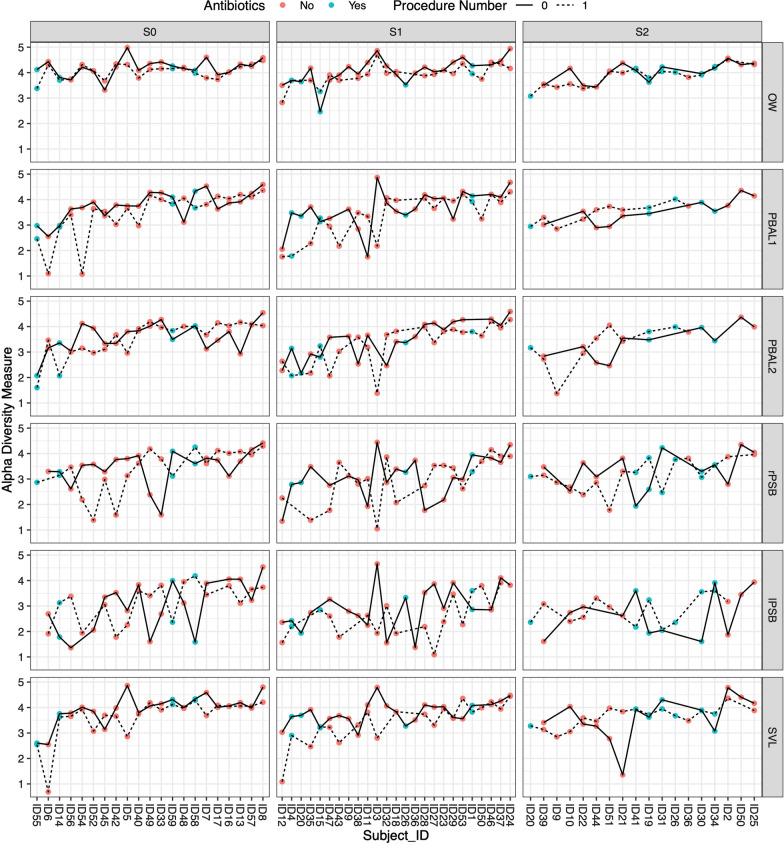
Alpha diversity as measured by the Shannon index, stratified by disease, sampling modality and whether subjects received antibiotics between the two procedures. The samples from the first bronchoscopy are connected by continuous lines, whereas the second bronchoscopy samples are connected by a dotted line. Samples of individuals that received antibiotics between the two procedures are coloured blue, subjects that did not receive antibiotics beween procedures are colored red. S0: control subjects, S1: COPD, FEV1 > 50% of predicted, S2: COPD, FEV1 < 50% of predicted. OW: oral wash. PBAL1: First fraction of protected broncho-alveolar lavage. PBAL2: second fraction of protected broncho-alveolar lavage. rPSB: protected specimen brush from right lower lobe

Alpha diversity was measured by the Shannon index, where a higher value indicates a more diverse sample. In statistical analyses, we did find that alpha diversity was lower in exam 2 than exam 1 for OW (*p* < 0.01, Wilcoxon signed rank test) and lower for pBAL1 (*p* = 0.054, Wilcoxon signed rank test). When we stratified by prescription of antibiotics between the procedures, the differences between OW samples (exam 1 vs 2) were only significant for subjects that had not received antibiotics. In bivariate regression analyses (linear regression, outcome difference in Shannon index between second and first bronchoscopy) we found no significant predictors. Figure 3 shows the alpha diversity for all participants at the two first procedures.

**Fig. 4:**
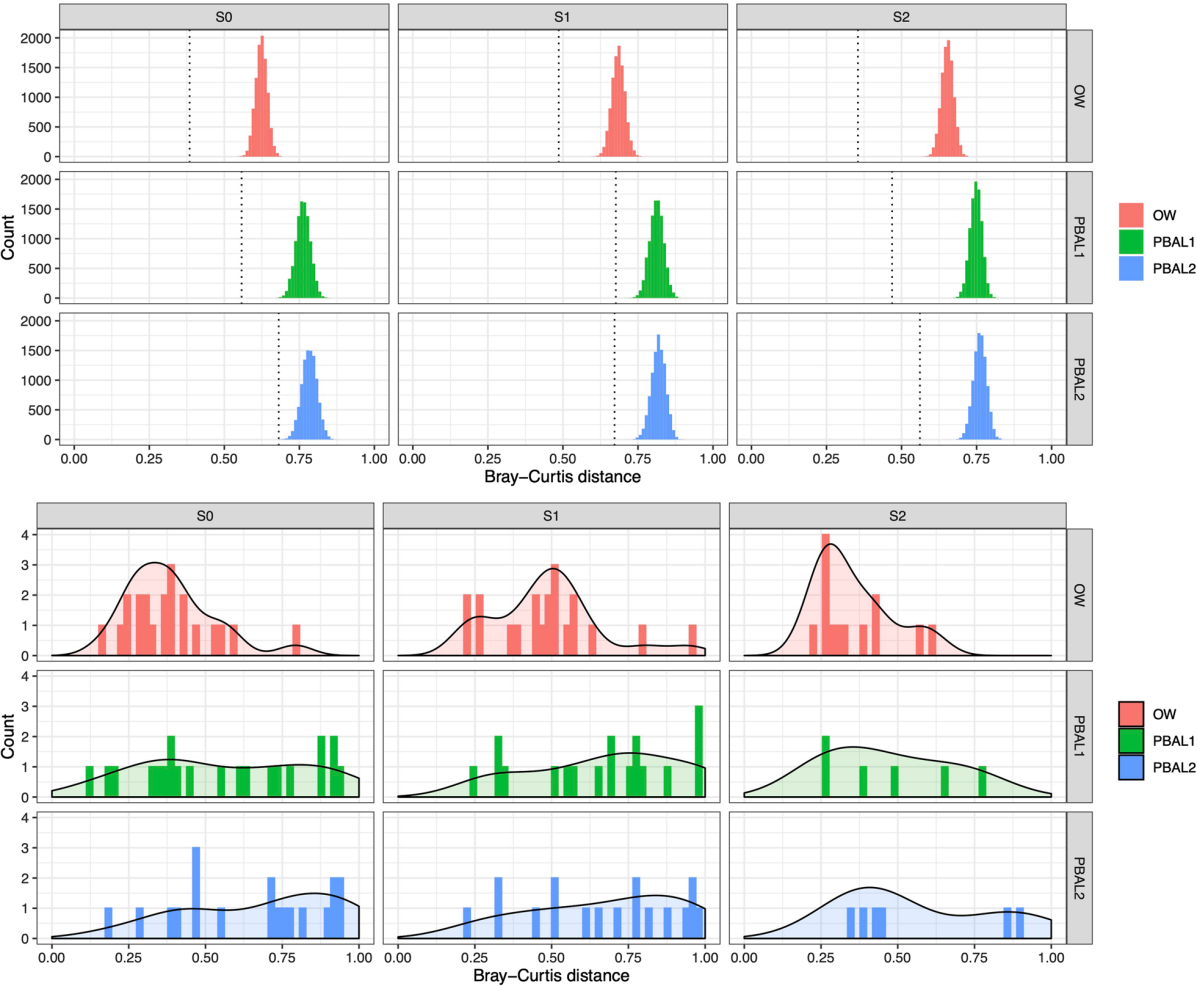
Top panels show mean Bray–Curtis distances between first and second bronchoscopy shown for oral wash (OW) and first fraction of the protected broncho-alveolar lavage samples (PBAL1) samples. The bars show the mean results of 10,000 permutations where random pairs of first and second bronchoscopy are compared. The dotted lines show the actual Bray–Curtis distances within individuals. The bottom panel show the actual distribution of the study data. Results are stratified by disease status: S0—no COPD, S1—COPD, FEV1 > 50%, S2—COPD, FEV1 < 50%

For beta diversity we compared the pairwise Bray–Curtis distance between the first and second procedure. With this distance measure, completely different composition between two samples is valued 1, whereas identical samples are valued 0. We performed a permutational test where we randomly marked pairs of samples (OW and PBAL1) as belonging to the same individual. Next, we compared the beta diversity of these random pairs with those of the actual pairs, to evaluate whether the beta diversity between procedure 1 and 2 for an individual would be lower than what could be expected by chance. The results of these tests are shown in Fig. 4. For both controls and COPD patients the observed values were lower than the permuted ones, indicating similarity beyond chance between samples from the same individual (10,000 simulations, thus *p* < 0.0001). Additional file 1: Fig. S3a and b shows similar figures for PBAL2 and rPSB compared to OW.

**Fig. 5:**
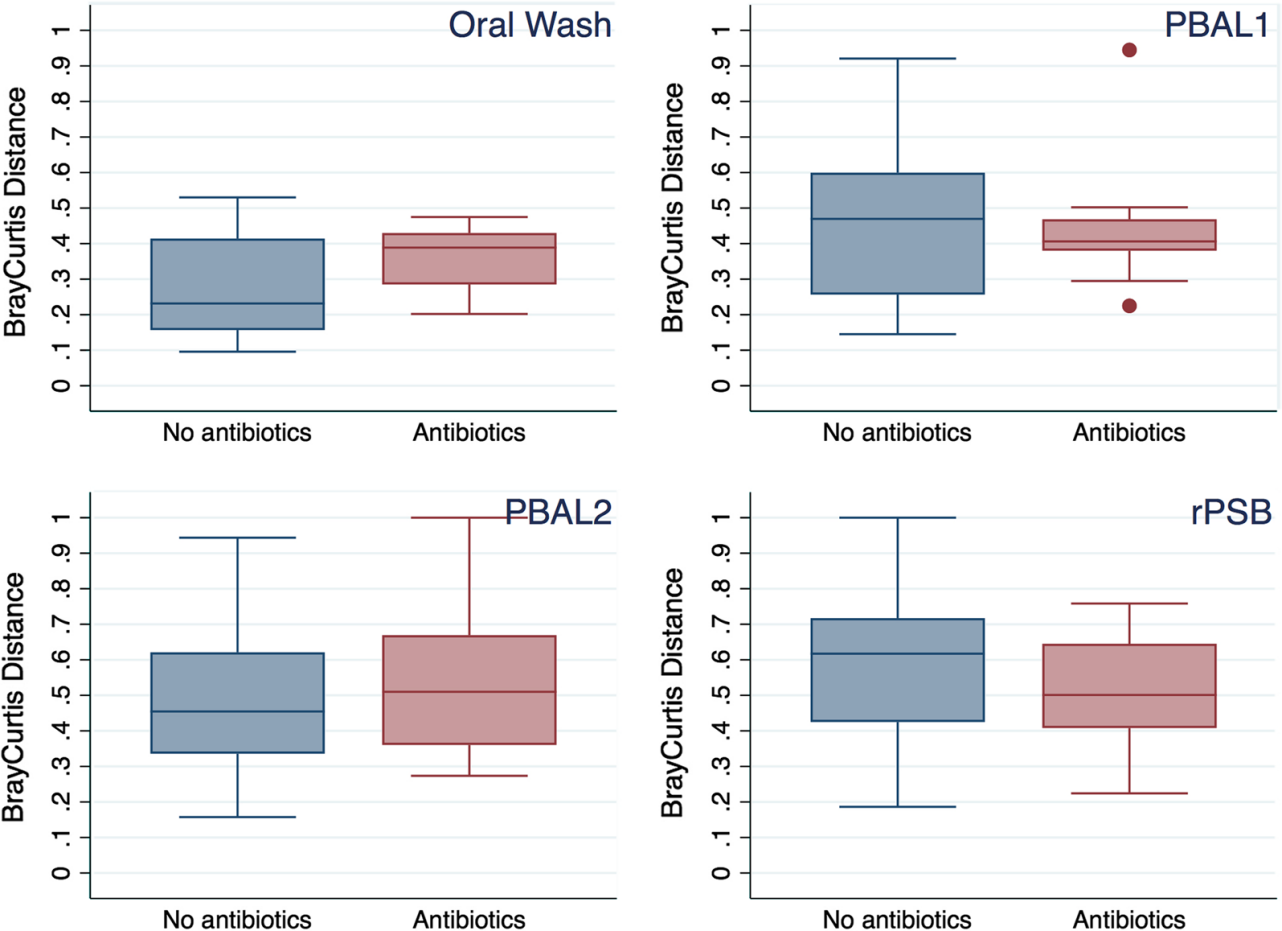
Bray–Curtis distances between first and second procedure in four sampling modalities (paired, within individuals). Stratified by whether subjects received antibiotics between the two procedures. PBAL1: first fraction of protected broncho-alveolar lavage. PBAL2: second fraction of protected broncho-alveolar lavage. rPSB: protected specimen brush from right lower lobe

We also looked at the impact of receiving antibiotics between exam 1 and 2, and the number of days between procedures. The beta-diversity measured by the Bray–Curtis distance in effect shows the difference in diversity between exam 1 and 2 (Fig. 5). The median values (value; IQR) for these distances increased in the order OW (0.28; 0.25), PBAL1 (0.45;0.27), PBAL2 (0.46;0.27) and rPSB (0.59; 0.31). When we compared these distances they all significantly differed (signed rank test, all comparisons *p* < 0.05). Bivariate beta regression analyses indicated increased diversity between procedures for subjects that received antibiotics between procedures (OW and PBAL2 samples, coefficients 0.401 and 0.83 and p-values 0.02 and 0.01, respectively). Bivariate beta regression analyses indicated no association between beta-diversity and number of days between procedures.

**Fig. 6:**
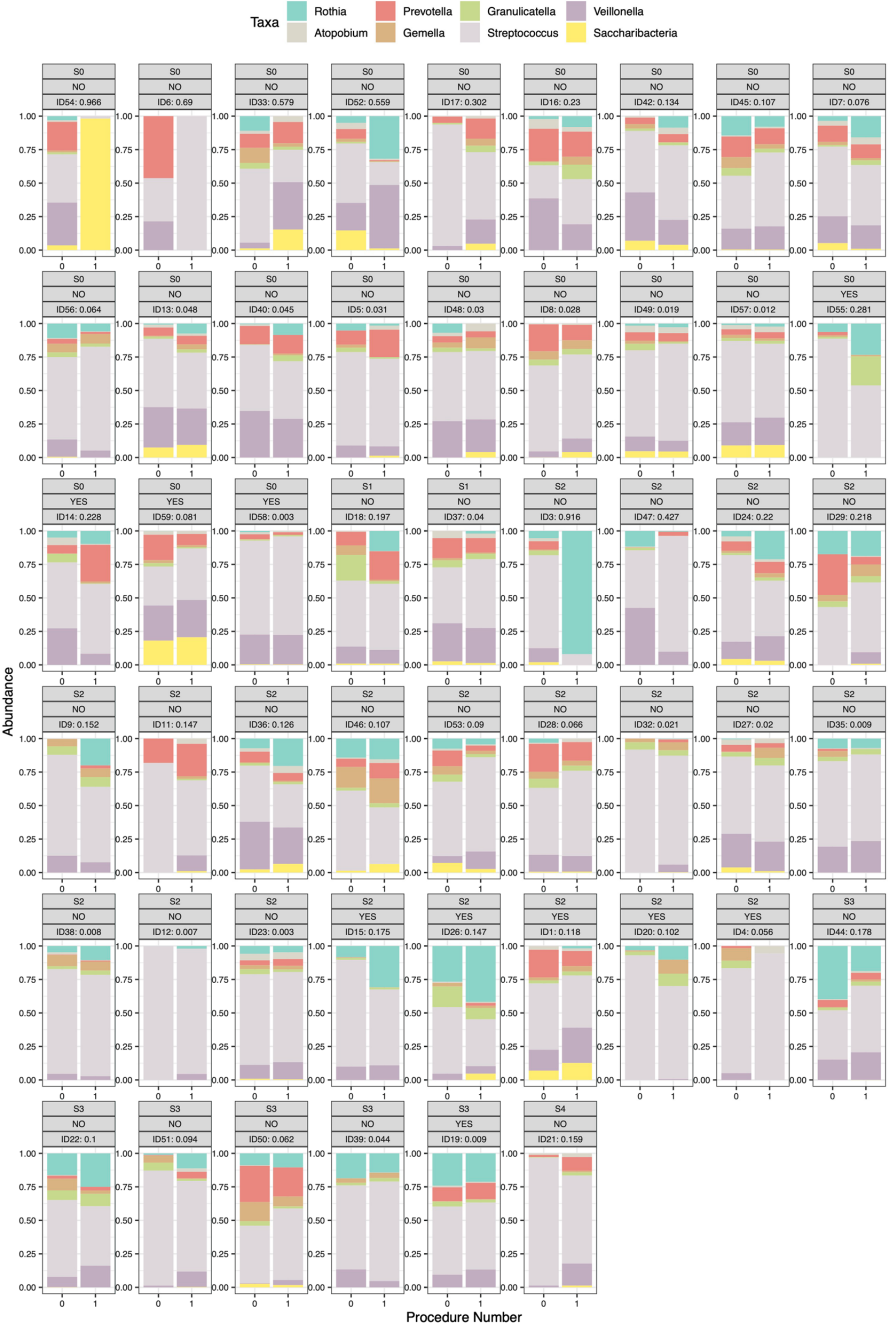
Taxonomic distribution of top 1% of amplicon sequence variants (ASVs) at genus level in first fraction of protected bronchoalveolar lavage samples (PBAL1), for all control subjects and participants with COPD. Ordered by Yue-Clayton dissimilarity index. Top boxes: Yue-Clayton dissimilarity index, exacerbation status (yes/no), and disease status (S0—controls, S1—COPD, FEV1 > 80% of predicted, S2—COPD, FEV1 50–80% of predicted, S3—COPD, FEV1 30–50% of predicted, S4—COPD, FEV < 30% of predicted). COPD—chronic obstructive pulmonary disease. FEV1—forced expiratory volume in 1 s

Finally, we combined the taxonomic presentation with the beta diversity analyses and looked at the ASVs that constituted more than 1% of the total number of sequences. Figure 6 shows the taxonomic distribution at the genus level for all the paired exams for the PBAL1 sorted by the beta-diversity measure Yue-Clayton index, which previously has been used to examine similarity in paired samples of the lung microbiome [18, 19]. The previously used threshold of 0.2 would indicate that 12 of the samples changed substantially. Additional file 1: Fig. S4a–c, show that the corresponding numbers are 17, 22 and 29 for OW, PBAL2 and rPSB, respectively.

Additional file 1: Fig. S5a and b shows the taxonomic distribution at the phylum and genus level of individuals with three procedures, showing the same pattern of stability as seen in subjects with two procedures.

## Discussion

These analyses from repeated bronchoscopies showed that the lower airway microbiota vary over time, and more so in the second BAL fraction and protected specimen brushes, than the first fraction of protected BAL. But, there were also distinct within-person similarities, perhaps pointing to a more stable part or fraction of the individual airway microbiota.

To our knowledge, this is the first report on the stability of the airway microbiome derived from repeated bronchoscopies in an observational study. Three intervention studies have examined the effects of antibiotics [4], interferon gamma [6], and highly active anti retroviral therapy (HAART) on the microbiome [5], and were not aiming to present changes at the individual level. The study by Segal et al. was based on an RCT where azitromycin was given to smokers with emphysema [4]. There was a control group of 10 individuals, where no changes were seen in alpha diversity (Wilcoxon), beta diversity (procrustes) or taxonomy (LEfSE). Wang et al. studied the effect of IFN-gamma on the microbiome of BAL samples in 10 IPF-patients. All participants received the intervention [6]. No significant changes were detected in alpha/beta-diversity, and there was also little signal in their LEfSE-analyses. Finally, Twigg reported a study on the effect of HAART to HIV patients, and compared with baseline samples in a cohort of 22 subjects without HIV. However, repeated measurements were only available for those subjects that received the intervention [5].

Sinha and colleagues investigated the variability of the microbiome in sputum samples at intervals of 2 days and 9 months in, respectively, 4 and 9 COPD patients [8]. Although obvious variation in both diversity and composition was observed, the authors concluded that they could demonstrate short time stability and a larger variability as sampling interval increased. However, the sample size was very low, and differences would have to be considerable to reach statistical significance. The increased variability with time could also be a result of for instance altered environmental (laboratory) contamination during sampling. Finally, no negative controls were sequenced, and no measures were taken to bioinformatically detect contaminant OTUs.

Mayhew and colleagues had a larger sample size with sputum samples from 101 COPD patients sampled in both stable and exacerbation states [7]. As in our study, they found that beta diversity within an individual was lower than between different individuals. However, they did not find significant changes in diversity from stable condition to exacerbation within the same individual, but in subjects with more exacerbations the microbiome seemed to be more unstable.

In the current study, we saw a trend that the diversity between the two procedures was higher in the airway samples than in the oral samples, and more so in the protected brush samples than in the BAL samples. This might be due to the airways microbiota being more transient than the oral microbiota, but could also be a result of the low-biomass nature of the lower airways that could make sampling more susceptible to random variation. Intercurrent events led to no consistent effect on diversity between procedures, but the heterogeneity of the population and also the heterogeneous nature of these events might have made it hard to trace such effects.

The current study adds to the existing knowledge by showing a degree of microbiome stability both in COPD patients and in individuals without known lung disease. A recently proposed hypothesis by Dickson et al., suggest the airway microbiota is the result of a constant influx and clearance, rather than a stable lung-residing microbiota [20]. Our findings represents a nuanced view, where albeit there is indeed great variability in time supporting the changing hypothesis, there are also signs of a small stable residing microbiome in the lower airways.

As in other studies, samples were dominated by *Firmicutes* ASVs as well as *Actinobacteria*, *Bacteriodetes* and *Proteobacteriae* [21–25]. At the genus level, the dominating ASVs were *Streptococci*, *Veillonella*, *Prevotella*, *Rothia* and *Haemophilus*, which also bears resemblance to observations made by previous authors [22, 23, 26]. No significance testing was made on the differences between COPD cases and controls in taxonomy analyses, as the full dataset of the MicroCOPD study including 249 study subjects, will provide better power.

This is the only airways microbiota study that has had as a primary objective to examine within-individual variation over time. The study was well powered with 131 bronchoscopies, given additional statistical power by the paired analyses. Both procedure and laboratory contamination were handled by using protected BAL and protected specimen brushes, as well as extensive negative control sampling and application of bioinformatic tools to identify potentially contaminating sequences.

Nevertheless, some methodological weaknesses deserve mentioning. First of all, the variation in sampling interval was considerable, making time a potential bias to the comparison. In our multivariate analyses of diversity, we could not detect an effect of adding the length of this interval as a covariate. Also, the ideal time interval is unknown, but the range of days between the first and second examination was from 88 to 349 days, and at least implies some degree of long-term stability. Second, it might be premature to draw firm conclusions based on analyses of only two timepoints in 62 individuals. But as far as we have been able to find, this is by far the largest repeated bronchoscopy study of the airways microbiome. Third, no consensus exist to date on when a difference in microbial composition between any two samples is factually clinically or statistically substantially different. Fourth, we have applied quite strict filtering and contaminant criteria in our bioinformatic analyses, reducing the number of ASVs from more than 27,000 to 551 ASVs, to avoid spurious inclusion of sequencing errors. We did not have mock community included in the earlier sequencing runs, and could thus not benchmark this approach. However, we have based our approach on previous publications, and it does seem highly unlikely that samples from 60 individuals should encompass more than 20,000 different microbial entities. Finally, having measurements of bacterial load (e.g. quantitative PCR) would have enabled analyses by amount of bacterial DNA in the original samples. Due to logistical and financial restrictions, qPCR was only performed for a small number of participants in the MicroCOPD study, but these analyses did show that the bacterial load differed by sampling modality—with higher bacterial load in OW and BAL samples than the protected specimen brushes [1].

In conclusion, the airways microbiota seem to vary over time. However, there is compositional microbiota stability within a person beyond that of pure chance, pointing to the possible existence of an indivudual core airways-residing microbiota.

## Supplementary Information

**Additional file 1.** Supplementary analyses.

## Data Availability

The dataset supporting the conclusions of this article is available in the Dryad repository, https://doi.org/10.5061/dryad.r2280gbbf.
