# Supplementary material for: Repeated bronchoscopy in health and obstructive lung disease: is the airway microbiome stable?
Source: BMC Pulm Med. 2021 Nov 2;21:342. doi: 10.1186/s12890-021-01687-0 (PMC8561866; doi:10.1186/s12890-021-01687-0)
Supplement: Supplementary file 1 — Additional file 1. Supplementary analyses. [file 12890_2021_1687_MOESM1_ESM.docx]

**Additional file 1**

Repeated bronchoscopy in health and obstructive lung disease: Is the airway microbiome stable?

Rune Nielsen* ^1, 2^, Yaxin Xue* ^3^, Inge Jonassen^3^, Ingvild Haaland^1, 2^, Øyvind Kommedal^4^, Harald G Wiker^1,4^, Christine Drengenes^1^, Per S Bakke^1^, Tomas ML Eagan^1,2^

* Shared first authors.

Affiliations

1. Dept of Clinical Science, Faculty of Medicine, University of Bergen, Bergen, Norway.
2. Dept of Thoracic Medicine, Haukeland University Hospital, Bergen, Norway
3. Computational Biology Unit, Dept of Informatics, University of Bergen, Bergen, Norway.
4. Dept of Microbiology, Haukeland University Hospital, Bergen, Norway

Correspondence:

Rune Nielsen

Dept of Clinical Science, Faculty of Medicine, University of Bergen Postboks 7804, N-5020 Bergen, Norway. E-mail: rune.nielsen@uib.no

*Supplemental figure 1a: Taxonomic distribution (phylum level) of all amplicon sequence variants in the oral wash samples (OW), shown by participant and procedure number (first column first bronchoscopy, second column is from second bronchoscopy). Ordered by disease stage (first grey box, S0: control subjects, S1: COPD, FEV_1_>50% of predicted, S2: COPD, FEV_1_ < 50% of predicted) and whether subjects have received antibiotics between procedures (second grey box; No or Yes) and. The third grey box is an anonymous participant identification number.*

**

*Supplemental Figure 1b: Taxonomic distribution (phylum level) of all amplicon sequence variants in the second fraction of the protected broncho-alveolar lavage samples (PBAL2), shown by participant and procedure number (first column first bronchoscopy, second column is from second bronchoscopy). Ordered by disease stage (first grey box, S0: control subjects, S1: COPD, FEV_1_>50% of predicted, S2: COPD, FEV_1_ < 50% of predicted) and whether subjects have received antibiotics between procedures (second grey box; No or Yes) and. The third grey box is an anonymous participant identification number.*

*Supplemental figure 1c: Taxonomic distribution (phylum level) of all amplicon sequence variants in the right protected specimen brush samples (rPSB), shown by participant and procedure number (first column first bronchoscopy, second column is from second bronchoscopy). Ordered by disease stage (first grey box, S0: control subjects, S1: COPD, FEV_1_>50% of predicted, S2: COPD, FEV_1_ < 50% of predicted) and whether subjects have received antibiotics between procedures (second grey box; No or Yes) and. The third grey box is an anonymous participant identification number.*

**

*Supplemental figure 2a: Taxonomic distribution (genus level) of the 20 most common amplicon sequence variants in the first fraction of the protected broncho-alveolar lavage samples (PBAL1), shown by participant and procedure number. Ordered by disease stage (top grey box, S0: control subjects, S1: COPD, FEV_1_>50% of predicted, S2: COPD, FEV_1_ < 50% of predicted) and by whether subjects have received antibiotics between procedures (second grey box).*

**

*Supplemental figure 2b: Taxonomic distribution (genus level) of the 20 most common amplicon sequence variants in the oral wash samples (OW), shown by participant and procedure number. Ordered by disease stage (top grey box, S0: control subjects, S1: COPD, FEV_1_>50% of predicted, S2: COPD, FEV_1_ < 50% of predicted) and by whether subjects have received antibiotics between procedures (second grey box).*

**

*Supplemental figure 2c: Taxonomic distribution (genus level) of the 20 most common amplicon sequence variants in the second fraction of the protected broncho-alveolar lavage samples (PBAL2), shown by participant and procedure number. Ordered by disease stage (top grey box, S0: control subjects, S1: COPD, FEV_1_>50% of predicted, S2: COPD, FEV_1_ < 50% of predicted) and by whether subjects have received antibiotics between procedures (second grey box).*

**

*Supplemental figure 2d: Taxonomic distribution (genus level) of the 20 most common amplicon sequence variants in the right protected specimen brush samples (rPSB), shown by participant and procedure number. Ordered by disease stage (top grey box, S0: control subjects, S1: COPD, FEV_1_>50% of predicted, S2: COPD, FEV_1_ < 50% of predicted) and by whether subjects have received antibiotics between procedures (second grey box).*

**

*Supplemental figure 3a: Mean results of 10,000 permutations of Bray-Curtis distances within oral wash (OW) and second fraction of the protected broncho-alveolar lavage samples (PBAL2) samples (top panel, bars), compared to actual Bray-Curtis distances within individuals (top panel, stapled lines). Results are stratified by disease status: S0 – no COPD, S1 – COPD, FEV_1_ > 50%, S2 – COPD, FEV_1_ < 50%. The bottom panels show the actual distribution of the study data.*

*Supplemental figure 3b: Mean results of 10,000 permutations of Bray-Curtis distances within oral wash (OW) and the right protected specimen brush samples (rPSB) samples (top panel, bars), compared to actual Bray-Curtis distances within individuals (top panel, stapled lines). Results are stratified by disease status: S0 – no COPD, S1 – COPD, FEV_1_ > 50%, S2 – COPD, FEV_1_ < 50%. The bottom panels show the actual distribution of the study data.*

**

*Supplemental figure 4a: Taxonomic distribution of top 1% of amplicon sequence variants (ASVs) at genus level in oral wash samples (OW), for all control subjects and participants with COPD. Ordered by Yue-Clayton dissimilarity index. Top boxes: Yue-Clayton dissimilarity index, exacerbation status (yes/no), and disease status (S0 - controls, S1 – COPD, FEV1 >80% of predicted, S2 – COPD, FEV1 50-80% of predicted , S3 – COPD, FEV1 30 - 50% of predicted, S4 – COPD, FEV < 30% of predicted). COPD – chronic obstructive pulmonary disease. FEV1 – forced expiratory volume in 1 second.*

*Supplemental figure 4b: Taxonomic distribution of top 1% of amplicon sequence variants (ASVs) at genus level in second fraction of protected bronchoalveolar lavage samples (PBAL2), for all control subjects and participants with COPD. Ordered by Yue-Clayton dissimilarity index. Top boxes: Yue-Clayton dissimilarity index, exacerbation status (yes/no), and disease status (S0 - controls, S1 – COPD, FEV1 >80% of predicted, S2 – COPD, FEV1 50-80% of predicted , S3 – COPD, FEV1 30 - 50% of predicted, S4 – COPD, FEV < 30% of predicted). COPD – chronic obstructive pulmonary disease. FEV1 – forced expiratory volume in 1 second.*

**

*Supplemental figure 4c: Taxonomic distribution of top 1% of amplicon sequence variants (ASVs) at genus level in right lower lobe protected specimen brush samples (rPSB), for all control subjects and participants with COPD. Ordered by Yue-Clayton dissimilarity index. Top boxes: Yue-Clayton dissimilarity index, exacerbation status (yes/no), and disease status (S0 - controls, S1 – COPD, FEV1 >80% of predicted, S2 – COPD, FEV1 50-80% of predicted , S3 – COPD, FEV1 30 - 50% of predicted, S4 – COPD, FEV < 30% of predicted). COPD – chronic obstructive pulmonary disease. FEV1 – forced expiratory volume in 1 second.*

*Supplemental figure 5a: Taxonomic distribution (phylum level) of all amplicon sequence variants in the first fraction of the protected broncho-alveolar lavage samples (PBAL1), shown by participant and procedure number (first column first bronchoscopy, second column second bronchoscopy, third column third bronchoscopy). Ordered by disease stage (first grey box, S0: control subjects, S1: COPD, FEV_1_>50% of predicted, S2: COPD, FEV_1_ < 50% of predicted) whether subjects have received antibiotics between two first procedures (second grey box; No or Yes). The third grey box is an anonymous participant identification number.*

*Supplemental figure 5b: Taxonomic distribution (genus level) of the 20 most common amplicon sequence variants in the first fraction of the protected broncho-alveolar lavage samples (PBAL1), shown by participant and procedure number. Ordered by disease stage (top grey box, S0: control subjects, S1: COPD, FEV_1_>50% of predicted, S2: COPD, FEV_1_ < 50% of predicted) and by whether subjects have received antibiotics between the two first procedures (second grey box).*
